# The influence of core self-evaluation of college students majoring in physical education on career decision-making difficulties: a chain mediation model

**DOI:** 10.3389/fpsyg.2025.1650716

**Published:** 2026-01-14

**Authors:** Chao Wang, Yaqi Li, Hongbo Zhao

**Affiliations:** 1 Dalian University of Science and Technology, Dalian, China; 2 School of Physical Education, Liaoning Normal University, Dalian, China

**Keywords:** physical education major, core self-evaluation, career decision-making difficulties, social support, career adaptability

## Abstract

**Background:**

This study aims to explore the relationship between core self-evaluation and career decision-making difficulties among undergraduate students majoring in physical education. It specifically examines the mediating roles of social support and career adaptability, as well as the chained mediating effect formed by these two factors.

**Methods:**

Using convenience sampling, 522 undergraduate physical education majors from six universities in Liaoning, Heilongjiang, and Hebei provinces were recruited. Data were collected via the Core Self-Evaluation Scale, Social Support Scale, Career Resilience Scale, and Career Decision Difficulty Scale. SPSS PROCESS macro was employed to analyze relationships among variables.

**Results:**

Core self-evaluation significantly and negatively predicted career decision-making difficulties. Social support and career adaptability each mediated the relationship between core self-evaluation and career decision-making difficulties. Furthermore, social support and career adaptability formed a chained mediating effect: core self-evaluation enhanced career adaptability by increasing social support, ultimately reducing career decision-making difficulties.

**Conclusion:**

Core self-evaluation influences career decision-making difficulties among physical education majors through both direct effects and indirect pathways mediated by social support and career adaptability.

**Discussion:**

Strengthening core self-evaluation, establishing specialized social support networks, and designing career adaptability training can alleviate career decision-making difficulties for physical education majors, providing concrete guidance for career counseling in higher education physical education programs.

## 1 Introduction

General Secretary Xi Jinping emphasized during the 14th collective study session of the Political Bureau of the Central Committee of the Communist Party of China (CPC) that to promote high-quality and full employment, we should insist on making the employment of college graduates and other young people groups the top priority (Xi, 2024). In recent years, the employment of college students in China has become a hot spot of concern for the whole society, and the difficulty of employment for college students is becoming more and more prominent (Li and Zhang, 2023). According to the Ministry of Education, the size of the 2024 national ordinary college graduates is expected to reach 11.79 million, an increase of 210,000 year-on-year, and competition in the job market continues to intensify (Liu and Zeng, 2024). As an important force in promoting the construction of a healthy China, college students majoring in sports are also facing a severe employment situation (Zhou and Wang, 2024). Due to the unique nature of their field, physical education majors face particularly pronounced challenges in career decision-making. These difficulties manifest primarily in two ways: First, there is a lack of clarity in self-assessment and career alignment. This refers to an unclear understanding of how their specialized skills-such as athletic abilities and teaching competencies-match various career requirements. For instance, they may struggle to determine whether their specialized athletic level meets professional athlete standards, or whether their teaching abilities suit the demands of different educational levels (Lu, 2025). Second, they face insufficient access to career information and inadequate planning. This includes a lack of understanding regarding policies within the sports industry and unclear direction in setting career goals. They are also prone to wavering under the influence of external opinions, which further complicates their career decision-making process (Yang et al., 2025).

Social ecosystem theory posits that individual development arises from interactions with ecosystems at different hierarchical levels. At its core, it encompasses micro-systems, meso-systems, and macro-systems, with all individual behaviors and choices embedded within these diverse social systems. Elements within these systems dynamically influence the individual development process (Qiao et al., 2025). Current sports majors face challenges such as intensified employment competition and diversified career paths, with their career decision-making difficulties stemming from multiple systemic factors. Core self-evaluation, as an individual's intrinsic perception of their abilities and value within the micro-system, when low, leads to insufficient decision-making confidence (Johnson et al., 2008) and negatively predicts career decision-making difficulties (Wu, 2015), Weak social support, as an individual's network of peripheral assistance and resources within the meso-level system, exacerbates difficulties in career decision-making (Zhou, 2021), Career adaptability, as an individual's dynamic professional adaptation competency within a microsystem, negatively predicts career decision-making difficulties (Jia, 2023), Career decision-making difficulties represent a professional predicament arising from the interaction between micro and meso systems. Their intensification may be associated with low core self-evaluation and insufficient career adaptability at the micro level, as well as weak social support at the meso level. While existing research on the employment of physical education majors in higher education institutions has yielded substantial findings, studies focusing on the factors influencing career decision-making difficulties remain relatively scarce. Against the backdrop of societal transformation, this study examines the impact mechanism of core self-evaluation on career decision-making difficulties. It aims to provide new theoretical foundations for alleviating such difficulties among physical education majors and enhancing their employment competitiveness. This research not only contributes to refining the theoretical framework for cultivating physical education professionals but also offers targeted guidance for university career counseling services.

### 1.1 The relationship between core self-esteem and career decision-making difficulties of college students majoring in physical education

Core self-evaluation refers to an individual's subjective judgment of his or her own efficacy, value, etc., and is also the basis for an individual's evaluation of other domains of his or her own (Judge et al., 1997), and individuals with high levels of core self-evaluation have more positive self-perceptions, are more adaptive and self-efficacious, and are able to positively regulate their own emotions (Judge et al., 1998). It has been shown that there is a significant positive correlation between the core self-evaluation of college students and the two dimensions and the total score of career value orientation, and the core self-evaluation can be used as one of the explanatory factors of career value orientation (Lu et al., 2017). The study also proved that there is a significant correlation between core self-evaluation and career decision-making difficulties, and that core self-evaluation is a significant negative predictor of career decision-making difficulties (Yang et al., 2025). Individuals with higher levels of core self-evaluation tend to have clear career self-exploration and career goal selection (Goldsmith et al., 1997), while individuals with lower levels of core self-evaluation show characteristics such as low self-esteem and discouragement, are prone to negative emotions, and are more susceptible to difficulties in career decision-making (Liu and Wang, 2016).

Therefore, Hypothesis H1 is proposed: core self-evaluation can negatively influence career decision-making difficulties.

### 1.2 The mediating role of social support

Social support refers to “the sum total of emotional, informational, and material help that a person receives and the socially connected people who provide it” (Cobb, 1976). College students' level of self-evaluation is significantly positively correlated with the level of social support they receive, and a positive view of people and events in their lives contributes to the formation of positive social awareness (Li, 2010). Research has shown that core self-evaluation of male inmates is significantly and positively correlated with perceptual social support (Yang and Wang, 2020), Individuals with lower core self-evaluations may have a more negative self-cognitive schema and, when faced with the same social support, may perceive less social support compared to individuals with high core self-evaluations (Lakey and Cassady, 1990), demonstrating that core self-evaluation positively predicts perceived social support (Li et al., 2022). When the level of social support is high, different groups are more confident in solving the difficulties they encounter through good social support (Li, 2020). Most people need some encouragement and support in the process of choosing a career or a career path, and it is only logical that an individual can make a major decision with the guidance of a support system (Meikle, 2008). At the same time, the study shows that the more social support an individual receives, the less difficulties he or she faces in the career decision-making process (Zhao, 2010), family support and other supports (teachers, classmates, friends) all affect the degree of career decision-making difficulties, thus showing individual differences in career decision-making difficulties (Li, 2012).

From this, it can be concluded that core self-evaluation may affect the career decision-making difficulties of college students majoring in physical education through social support, thus proposing the hypothesis H2 of this paper: social support can play a mediating role between core self-evaluation and career decision-making difficulties.

### 1.3 Mediating role of career resilience

Career adaptability refers to an individual's readiness for predictable career tasks, participation in career roles, and coping with career changes or unpredictable career problems in career situations (Savickas and Porfeli, 2012). Career resilience contains three characteristics: first, it is the ability to help individuals move forward; second, it is the ability that can be cultivated; and third, it is the result of the interaction between individuals and the environment (Zhao, 2011). Research has shown that essential traits of core self-evaluation, such as self-esteem, locus of control, and self-efficacy, are positively related to career adaptability (Yoo and Lee, 2019). Core self-evaluation positively predicts career adaptability and its individual dimensions (Zacher, 2014). When college students with high core self-evaluation can cope with career problems more confidently and calmly, which in turn improves their career resilience (Yang et al., 2021). It was found that career adaptability can directly affect career decision-making difficulties (Dong, 2017). Based on the research results about the association between career resilience and career exploration behavior, it can be seen that there is an obvious positive association between the two (Guan and Li, 2015). College students' career resilience is negatively correlated with career decision-making difficulties, and career resilience predicts career decision-making difficulties (Dong, 2017).

Based on the above obtained, hypothesis H3 is proposed: career resilience can play a mediating role between core self-evaluation and career decision-making difficulties.

### 1.4 Chain mediating role of social support and career resilience

It was found that social support positively contributes to college students' career resilience (Song et al., 2018), and a significant positive correlation between social support and career resilience (Li et al., 2023). When individuals perceive stronger social support, they are more likely to face challenges head on, accept change, remain resilient and increase their adaptability to the environment; whereas when social support is perceived to be lower, they have to take on more stress and insecurity in the face of adversity, which to a certain extent undermines the formation of career adaptability (Maggiori et al., 2013). Social support is an important factor influencing college students' career adaptability (Zhong and Ying, 2015), those college students who receive positive social support from social relationships such as parents, friends, and significant others have higher levels of career adaptability (Öztemel and Yildiz-Akyol, 2021).

Based on this, the present study proposed hypothesis H4: Social support and career resilience play a chain mediating role in the relationship between core self-evaluation and career decision-making difficulties.

**Figure 1:**
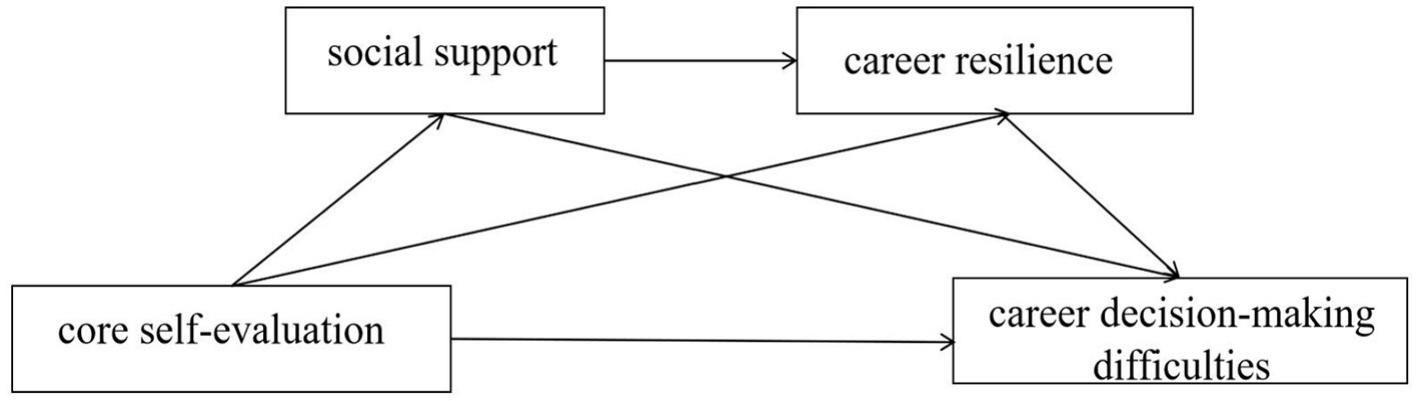
Chained intermediary hypothetical model.

To summarize, the present study, with college students majoring in physical education as the object of investigation, proposes the following hypotheses: (1) core self-evaluation negatively predicts career decision-making difficulties (H1); (2) social support mediates the relationship between core self-appraisal and career decision-making difficulties (H2); (3) career resilience mediates the relationship between core self-appraisal and career decision-making difficulties (H3); and (4) core self-appraisal through the social support and career resilience to chain mediate career decision-making difficulties (H4). Based on this, this study constructed a chain mediation model (Figure 1).

## 2 Methods

### 2.1 Statement of ethical approval

This study strictly adheres to the principle of “informed consent” outlined in the Declaration of Helsinki. The research protocol has been approved by the Institutional Review Board (IRB) of Liaoning Normal University (Approval Number: LL2025093; Approval Date: March 25, 2025). During data collection, anonymous questionnaires were employed to fully protect participant privacy. All participating students voluntarily signed informed consent forms after receiving full disclosure and retained the right to withdraw from the study at any time.

### 2.2 Subjects

To determine the appropriate sample size, the study employed G^*^Power 3.1 software for a pre-test power analysis. Based on a linear multiple regression model, with a medium effect size (f^2^ = 0.15), significance level (α = 0.05), and statistical power (1-β = 0.80), the calculation indicated that a minimum sample size of 82 participants was required to detect chained mediating effects.

Using convenience sampling, this study randomly selected nine universities (including normal universities, specialized sports universities, and comprehensive universities) in Liaoning Province, Heilongjiang Province, and Hebei Province (provinces with large populations and representative student bodies from across China). Online questionnaires were distributed, with a total of 553 questionnaires distributed. After strict screening of the recovered questionnaires and elimination of invalid questionnaires such as incomplete answers and confusing logic, 522 valid questionnaires were obtained, and the effective recovery rate of the questionnaires reached 94.4%. Among them, 258 (49.4%) were male students, 264 (50.6%) were female students, and the numbers of freshmen, sophomores, juniors, and seniors were 106 (20.3%), 140 (26.8%), 134 (25.7%), and 142 (27.2%) respectively.

### 2.3 Research instruments

#### 2.3.1 Core self-evaluation scale

We adopted the Core Self-Evaluation Scale, which was revised by Judge et al., optimized by Chinese scholars Du et al. (2012) and has a relatively universal scope of application in China. The scale is one-dimensional, with a total of 10 response items, (e.g., I believe I can achieve success in life). A five-point Likert scale was used, with higher total scores representing higher core self-evaluations. The Cronbach alpha coefficient of the scale in this study was 0.917, indicating that the scale has good reliability, which ensures that the data of the study are true and stable, and lays the foundation for the accuracy of the study. The factor loadings for each item are greater than or close to 0.50, indicating that the scale meets the criteria for structural validity.

#### 2.3.2 Career decision difficulty scale

We adopted the Career Decision-Making Difficulties Scale developed by Long and Du. This scale consists of 16 items, divided into 4 dimensions (Peng and Long, 2001). career self-exploration (e.g., I will learn about my own personality, interests, abilities, and values through other people's evaluations of me), career information exploration (e.g., I collect information about the job market and employment opportunities), career planning exploration (e.g., I participated in training or social practices that help with career planning), and career goal exploration (e.g., I have short-term career goals). Using the Likert five-point scale, all items are reverse-scored. Raw scores for reverse-scored items must first be converted—for example, 5 points become 1 point, 4 points become 2 points, and so on. The higher the total score, the greater the difficulty in making career decisions. A five-point Likert scale was used and all questions were reverse scored. The Cronbach alpha coefficient of this scale in this study was 0.943, which, with reference to psychometric standards, indicates that the scale is highly reliable and can provide reliable and stable data support for the study. The factor loadings for each item are all greater than or close to 0.50, indicating that the scale meets the criteria for structural validity.

#### 2.3.3 Social support scale

We adopted the most widely used Perceived Social Support Scale, which was developed by Zimet et al. and adapted by Jiang for localized research needs. This scale has demonstrated good reliability and validity (Jiang, 2001). The scale consists of 12 questions, including three dimensions: family support (e.g., my family can help me in a practical and concrete way), friend support (e.g., I can rely on my friends in times of trouble), and other people's support (e.g., I can share my joys and sorrows with some people), and it adopts a seven-point Likert scale, where the higher the score, the higher is the level of support the individual feels from his/her family, friends or others. The higher the score, the higher the level of support the individual feels from family, friends, or others. The Cronbach's alpha coefficient of the scale in this study is 0.930, which indicates that the scale has good reliability from the perspective of psychometrics and can guarantee reliable data. The factor loadings for each item are greater than or close to 0.50, the dimensionality aligns with the data characteristics, and the construct validity is sound.

#### 2.3.4 Career adaptability scale

For this study, we used the Chinese version of the Career Adapt-Abilities Scale, which was developed by Hou et al. (2012) based on the international version. The scale consists of four dimensions: Career Concern (e.g., I can think clearly about what my future looks like), Career Control (e.g., I can make decisions independently), Career Curiosity (e.g., I can explore and learn about my surroundings), and Career Self-Confidence (e.g., I can complete tasks efficiently), with a total of 24 questions. A five-point Likert scale was used, with higher scores indicating greater career resilience of the individual. The Cronbach's α coefficient of the scale in this study is 0.958, which confirms that the scale's reliability is up to standard, which can guarantee the quality of the data and help the study to advance accurately. The factor loadings for each item are all greater than or close to 0.50, indicating that the items effectively capture the dimensions they represent, thus establishing reliable construct validity.

### 2.4 Statistical processing

This study used SPSS 27.0 software as well as AMOS 24.0 software to analyze the data. The sample size of the questionnaire was also calculated through G^*^Power software, with the effect size set at 0.3 (Borenstein et al., 1990), significance set at 0.05, and statistical test power set at 0.8, which finally resulted in the required sample size of 82. This study first assessed data missingness, with an overall missingness rate of 5.9%. Given the random nature and extremely low proportion of missing values, observations containing missing data were directly deleted. No additional data cleaning was performed; only the theoretical range of 1-5 points for scale item values was verified, with no abnormal values detected. Ultimately, 522 samples free of missing data and logical errors were used for subsequent analysis.

## 3 Results

### 3.1 Common method bias test

Since this study used four measurement tools for the same subjects, and since the data were all derived from subjects' self-report, in order to effectively avoid the problem of common method bias, the Harman one-way test was used to conduct exploratory factor analysis on all the items when dealing with the data. The results showed that there were nine factors with eigenvalues greater than 1, and the explained variance of the first factor was only 37%, which was lower than the critical threshold of 40%, and thus it was judged that there was no serious common method bias in this study. Simultaneously, the four-variable, multi-factor CFA models all demonstrated good fit (χ^2^/df < 3, CFI > 0.9, RMSEA < 0.08), indicating that common method variance is manageable. It can be concluded that the common method variance in this study is relatively low and will not substantially impact the reliability of the research findings.

### 3.2 Descriptive statistics and correlation analysis

Using an independent samples *t*-test, we analyzed differences between male and female physical education majors in core self-evaluation, social support, career adaptability, and career decision-making difficulty. The Cohen's d value for social support was 0.14, while that for career decision-making difficulty was 0.27, both indicating small effects. The results indicate that the significantly higher scores among female students on the career decision-making difficulty dimension may be related to the long-standing occupational gender bias in the sports industry. Conversely, male students demonstrated higher levels of social support compared to female students, likely stemming from their more frequent interactions during team training and competitions, which fostered greater support networks, resulting in a significant difference (see Table 1).

**Table 1:** Gender differences across different variables (*n* = 522).

	**Sex**	**Number**	**M ±SD**	***F***	***p***
Core self-evaluation	Male	258	2.42 ± 0.55	0.277	0.000^**^
	Female	264	2.66 ± 0.60		
Social support	Male	258	2.08 ± 0.71	26.585	0.000^**^
	Female	264	1.99 ± 0.59		
Career resilience	Male	258	1.93 ± 0.60	13.917	0.000^**^
	Female	264	1.92 ± 0.49		
Difficulty in career decision making	Male	258	3.83 ± 0.64	9.917	0.002^**^
	Female	264	3.99 ± 0.55		

^*^P < 0.05, ^**^P < 0.01.

Using a one-way analysis of variance (ANOVA), this study compared differences among physical education majors across different academic years in core self-evaluation, social support, career adaptability, and career decision-making difficulties. Effect size analysis revealed that grade-level differences in career adaptability (η^2^ = 0.011) and core self-evaluation (η^2^ = 0.032) were both small effects. For the 12 independent comparisons across 4 variables × 3 grade groups, Holm-Bonferroni correction was applied at α = 0.05 to adjust Type I errors. Results indicated that all significant differences passed the test. The analysis results indicate that statistically significant differences exist across grade levels among physical education majors in the dimension of core self-evaluation. Freshmen exhibit the lowest level of core self-evaluation, while sophomores demonstrate the highest. Significant differences in social support levels are observed across grade levels, with sophomores receiving the least social support and freshmen receiving the most. No significant differences were found in career adaptability and career decision-making difficulties among students of different grade levels (see Table 2).

**Table 2:** Grade-level differences across different variables (*n* = 522).

	**Grade**	**Number**	***M ±SD***	***F***	***p***
Core self-evaluation	Freshman	106	2.43 ± 0.61	5.613	0.000^**^
	Sophomore	140	2.70 ± 0.52		
	Junior	134	2.54 ± 0.62		
	Senior	142	2.47 ± 0.58		
Social Support	Freshman	106	2.54 ± 0.59	0.937	0.042^*^
	Sophomore	140	1.99 ± 0.70		
	Junior	134	2.04 ± 0.54		
	Senior	142	2.00 ± 0.66		
Career resilience	Freshman	106	2.11 ± 0.71	1.943	0.122
	Sophomore	140	2.04 ± 0.65		
	Junior	134	1.89 ± 0.58		
	Senior	142	1.97 ± 0.41		
Difficulty in career decision making	Freshman	106	1.85 ± 0.53	1.389	0.245
	Sophomore	140	1.99 ± 0.65		
	Junior	134	1.93 ± 0.55		
	Senior	142	3.85 ± 0.66		

^*^P < 0.05,^**^P < 0.01.

The results of the correlation analysis showed (Table 3) that core self-evaluation was significantly positively correlated with social support (*r* = 0.551, *p* < 0.01) and also with career resilience (*r* = 0.619, *p* < 0.01), while it was significantly negatively correlated with career decision-making difficulties (*r* = −0.542, *p* < 0.01). There was a significant positive correlation between social support and career resilience (*r* = 0.735, *p* < 0.01) and a significant negative correlation with career decision-making difficulties (*r* = −0.742, *p* < 0.01). In addition, there was also a significant negative correlation between career resilience and career decision-making difficulties (*r* = −0.755, *p* < 0.01). These results indicate that there is a significant positive association between core self-evaluation, social support and career resilience, while all three are significantly negatively associated with career decision-making difficulties. This result provides preliminary support for the testing of the hypotheses of the subsequent study, and also lays the foundation for further exploration of the relationship between the variables.

**Table 3:** Correlation analysis between different variables (*n* = 522).

**Variables**	**M ±SD**	**1**	**2**	**3**	**4**
1 Core self-evaluation	2.54 ± 0.59	1			
2 Social Support	2.04 ± 0.65	0.551^**^	1		
3 Career resilience	1.92 ± 0.55	0.619^**^	0.735^**^	1	
4 Difficulty in career decision making	3.91 ± 0.60	−0.542^**^	−0.742^**^	−0.755^**^	1

^**^P < 0.01.

### 3.3 The problem of multicollinearity

Since all the variables are significantly correlated, the problem of multicollinearity may exist. Therefore, covariance diagnosis was performed by standardized analysis in this study. The results show that the variance inflation factor values (VIF) 1.673~2.535 of each predictor variable are less than 5, tolerance 0.394-0.598, condition index < 30. Therefore, the data do not have serious multi collinearity (Wen et al., 2018), which can be further tested for chained mediation effects.

### 3.4 Structural equation modeling

Prior to conducting structural equation modeling analysis, this study tested assumptions such as data normality and linear relationships. Results indicate that the data meet model applicability requirements. Using AMOS 26.0, the modeling was conducted in two stages: First, the measurement model for the four latent variables was validated. Criteria of χ^2^/df < 3, GFI ≥ 0.90, and RMSEA < 0.08 were met for all variables (e.g., Core Self-Evaluation: χ^2^/df = 2.705, CFI = 0.987; Career Adaptability χ^2^/df = 2.320, RMSEA = 0.050), confirming acceptable reliability and validity. Subsequently, an initial structural model incorporating three mediating pathways and controlling for gender and grade was constructed. The fit indices (χ^2^/df < 5, CFI > 0.90, RMSEA < 0.08) met the criteria, indicating good model fit and enabling direct use for mediating effect testing.

**Figure 2:**
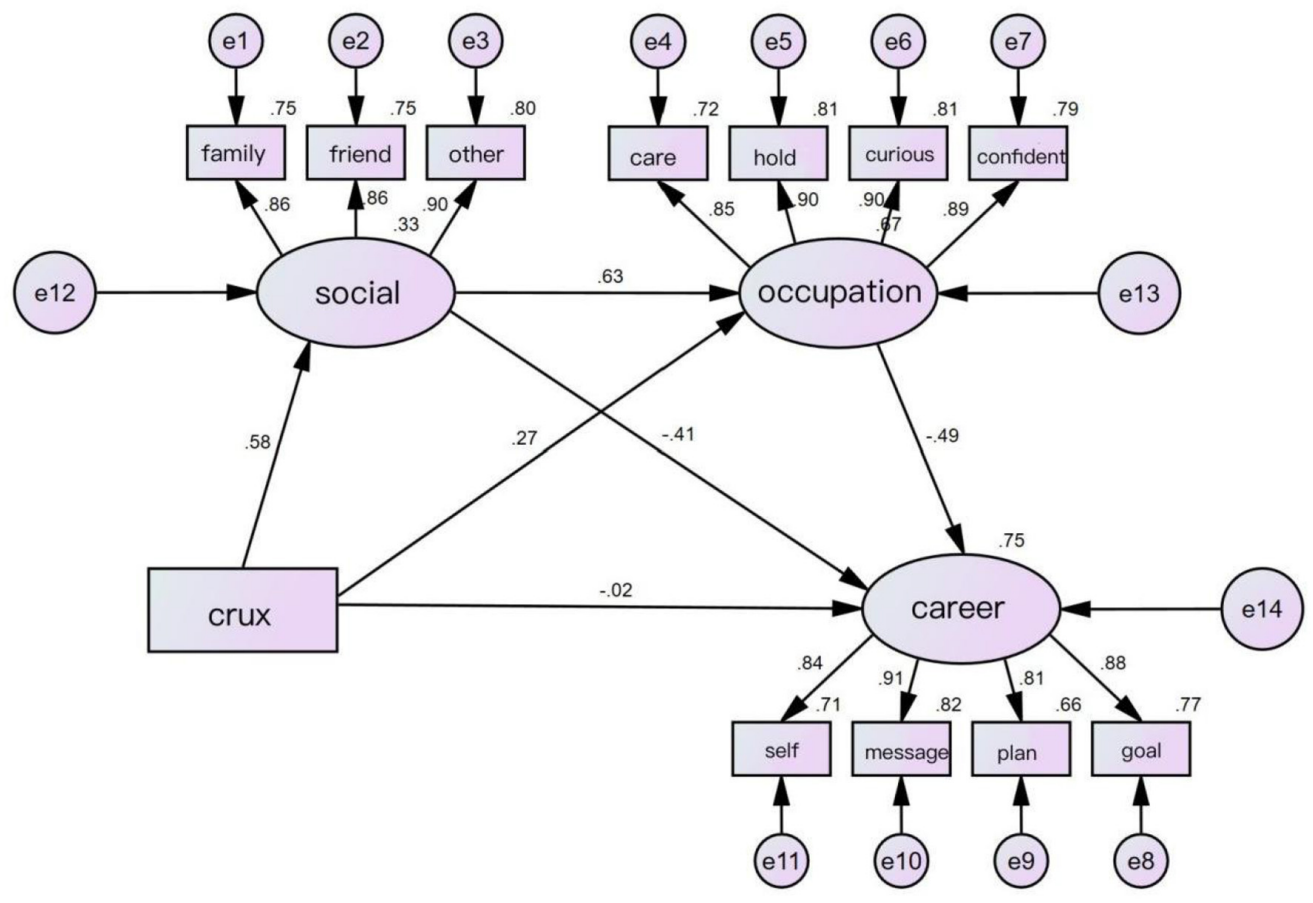
SEM model analysis of intermediation.

Amos 24.0 was used to test the fit of the measurement model to the actual data. As shown in Table 4, X2/df = 3.773 < 5, which is generally considered an acceptable model fit.GFI = 0.942, NFI = 0.968, and CFI = 0.976, which are all greater than 0.9 indicate that the model has a good validity of fit, and RMSEA = 0.073 < 0.08 indicates that the fit to the data is acceptable. In summary, the measurement model meets the desired criteria (Xiong and Song, 2019), the structural model can be further tested. As shown in Figure 2, core self-evaluation positively predicted social support (β = 0.58, *p* < 0.001), and core self-evaluation had a significant positive predictive effect on career resilience (β = 0.27, *p* < 0.001) while showing a significant negative predictive effect on career decision-making difficulties (β = −0.02, *p* < 0.001). Social support showed a significant positive predictive effect on career resilience (β = 0.63, *p* < 0.001), while significantly negatively predicting career decision-making difficulties (β = −0.41, *p* < 0.001). Career resilience was a significant negative predictor of career decision-making difficulty (β = −0.49, *p* < 0.001), suggesting that the higher the level of an individual's career resilience, the lower the level of career decision-making difficulty.

**Table 4:** Fit indices for the mediating role model of social support and career resilience.

***X*^2^/df**	**GFI**	**NFI**	**IFI**	**TLI**	**CFI**	**RMSEA**
3.773	0.942	0.968	0.976	0.968	0.976	0.073

### 3.5 Mediating effects test

Table 5 presents the results of the mediation effect analysis of social support and career resilience between core self-evaluation and career decision-making difficulties.

**Table 5:** Analysis of the mediating effects of social support and career resilience on core self-ratings and career decision-making difficulties.

**路径**	**Effective quantity**	**SE**	**P**	**95%CI**	
				**LB**	**UB**
Ind1: Core self-evaluation → social support → career decision-making difficulties	−0.234	0.039	0.000	−0.313	−0.163
Ind2: Core self-evaluation → career resilience → career decision-making difficulties	−0.134	0.029	0.000	−0.198	−0.081
Ind3: Core self-evaluation → social support → career resilience → career decision-making difficulties	−0.181	0.027	0.000	−0.236	−0.129

This study employed the Bootstrap method (with 5,000 repeated samples) to test the significance of the mediating effects, with the core criterion being whether the 95% confidence interval (CI) of the mediating path included zero. As shown in Table 5, the 95% CIs for all three mediating paths did not include zero. Ind1: [-0.313,−0.163], Ind2: [-0.198,−0.081], Ind3: [-0.236,−0.129], and the corresponding p-values for each path effect were all 0.000, fully validating the mediating effects. Furthermore, the potential influence of omitted confounding factors must be considered. For instance, family occupational background may affect students' career cognition and social support networks, while regional disparities in sports employment market supply and demand may impact the actual cultivation of career adaptability. These variables, not included in the model, may exert subtle interference on the mediating paths by indirectly affecting the acquisition of social support and the cultivation of career adaptability.

## 4 Discussion

First, core self-evaluation negatively predicts career decision-making difficulties, which is consistent with previous research (Judge et al., 1997). Individuals with higher core self-evaluations are usually accompanied by stronger self-confidence and self-efficacy, which enables them to take a more positive stance when faced with challenges in career decision-making. This positive psychological trait makes them less confused and anxious when facing career choices, thus reducing the degree of difficulty in career decision-making. In addition, individuals with low core self-evaluation tend to be poor at setting clear goals and taking action, which further contributes to their uncertainty and procrastination behaviors in the career decision-making process. Increasing the level of students' core self-evaluation can reduce career decision-making difficulties, and at the same time expand the ways to transform negative academic emotions into positive ones by stimulating positive academic emotions in students (Zhou, 2021; Lu et al., 2017). Core self-evaluation is not only an important predictor of career decision-making difficulties, but also provides an important theoretical basis for career counseling. This provides practical evidence for universities to optimize career guidance for physical education majors and improve talent cultivation, thereby alleviating their employment challenges.

Second, social support mediated the relationship between core self-evaluation and career decision-making difficulties. Core self-evaluation positively predicts social support and social support negatively predicts career decision-making difficulties, which is consistent with previous studies (Yang and Wang, 2020). In social support buffering theory, social support can help individuals better cope with stressful situations by providing emotional, informational, and instrumental support (Cohen and Wills, 1985). For career decision-making, support from family, friends, and mentors can increase an individual's confidence, provide valuable career information, and help alleviate anxiety during the decision-making process. Individuals with high core self-evaluations are usually better at utilizing social support resources, which further reduces career decision-making difficulties. This suggests that in career counseling, attention should be paid to cultivating individuals' social support networks, especially through the cooperation between families and schools, to provide more career guidance and emotional support for college students. By establishing a home-school career guidance collaboration platform that integrates parental experience sharing with professional school guidance, we can provide college students with diverse career advice and emotional support, thereby strengthening the development of their social support networks.

Once again, career resilience played a mediating role in the process of core self-evaluation influencing career decision-making difficulties. Core self-evaluation positively predicted career resilience and career resilience negatively predicted career decision-making difficulties, validating previous studies (Zhao, 2011; Zacher, 2014). Career construct theory suggests that career resilience reflects an individual's ability to adapt in the face of career changes and challenges (Savickas, 1997), individuals with high core self-evaluations usually have stronger self-regulation and goal orientation, which enable them to better adapt to changes in the career environment and actively cope with uncertainty in career decision-making. As a dynamic psychological resource, career resilience can help individuals maintain flexibility and initiative in the process of career exploration and decision-making, thus reducing career decision-making difficulties. Therefore, enhancing individual career resilience should be an important goal of career counseling, for example, through career planning courses and practical activities to help college students enhance their career resilience. Therefore, enhancing individuals' career adaptability should be a key objective of career counseling. For instance, through career planning courses and practical activities, college students can be assisted in strengthening their professional adaptability.

Finally, the study concluded that social support and career resilience play a chain mediating role between core self-evaluation and career decision-making difficulties. Within the context of Chinese cultural background, the chain-like mediating effect of social support and career resilience between core self-evaluation and career decision-making difficulties aligns with the cultural emphasis on interpersonal support in Chinese society. It also resonates with the traditional cultural value of cultivating oneself, accumulating potential, and advancing to achieve goals, further highlighting the applicability of this mechanism within the Chinese cultural context (Zhang, 2023). This result suggests that core self-evaluation not only directly affects career decision-making difficulties, but also indirectly reduces career decision-making difficulties by increasing social support and improving career resilience (Lakey and Cassady, 1990; Yang et al., 2021). Specifically, core self-evaluation enhances individuals‘ self-perception, social support provides individuals with emotional and informational resources, and individuals with high career resilience are able to proactively search for information, analyze problems, and flexibly adjust their decision-making strategies, which helps them enhance their adaptive ability to the career environment, and thus cope with the challenges in career decision-making more effectively. This chain mediation further reveals the synergistic effect of social support and career resilience in the career decision-making process. In career counseling, emphasis should be placed on simultaneously enhancing individuals' social support levels and career resilience, for example, through group counseling and mentoring programs, to help college students build support networks and enhance career resilience, thereby reducing career decision-making difficulties more effectively.

## 5 Research insights and shortcomings

The present study demonstrates that social support and career resilience play a chain mediating role in the core self-evaluation on career decision-making difficulties of college students majoring in physical education, which provides important theoretical and practical insights for the career development of college students majoring in physical education. First, this study confirms that core self-evaluation can act directly on career decision-making difficulties, and at the same time introduces social support and career resilience as mediating variables to enrich the theoretical framework in the field of career decision-making. The results of the study indicate that enhancing individuals' core self-evaluation level can effectively alleviate career decision-making difficulties, while social support and career resilience play an important bridging role in this process. Secondly, for educators in colleges and universities, they should focus on cultivating the core self-evaluation ability of college students majoring in physical education, and help students establish positive self-perception through mental health education and career planning courses. At the same time, colleges and universities should strengthen the construction of social support system, provide students with more career guidance and resource support, and enhance students' career decision-making ability through career resilience training. In addition, families and society should also actively participate in creating a favorable external support environment for students.

Despite achieving certain results, this study has several limitations. First, inadequate control of confounding variables—such as socioeconomic status (SES) and mental health factors—may have overestimated the predictive effect of core self-evaluation on social support and its mediating effect on career adaptability. Future research should incorporate family SES and occupational anxiety into the model to enhance explanatory precision. Second, although common method bias was demonstrated to be acceptable through pre-control and post-verification, self-reported data may exhibit social desirability bias, causing minor interference in the chained mediation path. Finally, the sample lacks sufficient representativeness and breadth. The sample primarily consists of physical education majors from universities in Liaoning, Heilongjiang, and Hebei provinces. Future studies should broaden the sample scope to include more regions and different types of universities, thereby enhancing the generalizability of findings. This cross-sectional study cannot reveal the dynamic relationships between core self-evaluation, social support, career adaptability, and career decision-making difficulties over time. Future research should employ longitudinal tracking designs to delve deeper into the interactive mechanisms of these variables over time.

In summary, this study preliminarily reveals the chain-mediated mechanism through which core self-evaluation influences career decision-making difficulties among physical education majors. However, its limitations suggest that subsequent research should further optimize variable control, data collection, and sample design. Through more rigorous future designs, the formation mechanism of career decision-making difficulties among physical education students can be more precisely analyzed, providing robust academic support for universities to develop differentiated career counseling strategies and thereby promote their healthy career development.

## Data Availability

The raw data supporting the conclusions of this article will be made available by the authors, without undue reservation.
